# Long-term survival of subglottic adenoid cystic carcinoma: a case report

**DOI:** 10.3389/fonc.2025.1611107

**Published:** 2025-07-28

**Authors:** Yu-Ting Yin, Chao Gui

**Affiliations:** ^1^ Department of Gastrointestinal Surgery, Hubei Cancer Hospital, Tongji Medical College, Huazhong University of Science and Technology, Hubei, Wuhan, China; ^2^ Department of Head and Neck Surgery, Hubei Cancer Hospital, Tongji Medical College, Huazhong University of Science and Technology, Hubei, Wuhan, China

**Keywords:** adenoid cystic carcinoma, surgery, recurrence, radiotherapy, case report

## Abstract

Adenoid cystic carcinoma of the larynx is rare, mainly localizing in the subglottic region. The clinical presentation is predominantly characterized by dyspnea and hoarseness. In this study, a 38-year-old woman presented symptoms that included hoarseness, wheezing, and coughing. A diagnosis of subglottic adenoid cystic carcinoma was made by laryngoscopy and biopsy and a total laryngectomy was subsequently performed. A decade after the initial surgical intervention, the tumor recurred in the local region. Consequently, a subsequent surgical procedure was undertaken, accompanied by postoperative radiotherapy. A subsequent 5-year follow-up examination revealed no evidence of local recurrence or distant metastasis. Subglottic adenoid cystic carcinoma is an uncommon malignancy. A comprehensive surgical excision can yield a favorable therapeutic outcome. In cases of recurrence, subsequent surgical intervention and postoperative supplemental radiotherapy can achieve positive results.

## 1 Introduction

Adenoid cystic carcinoma (ACC) is a rare type of cancer that accounts for 1.5–2% of all head and neck cancers (1). Its main occurrence is in the major and minor salivary glands. Tumors of the minor salivary glands are found most frequently in the oral cavity, especially the hard palate, and less commonly in the nasal cavity, sinuses, pharynx, and larynx (2). In the larynx, minor salivary gland tumors are rare and account for <1% of laryngeal tumors, whereas most of the minor salivary glands are concentrated under the vocal folds (3).

ACC is characterized by gradual growth, aggressive behavior, and high likelihood of local recurrence and distant metastasis (4). Main clinical symptoms manifest as dyspnea, hoarseness, coughing, blood in the sputum, and other discomforts. The neurophilic and infiltrative nature of ACC makes it difficult to find an optimal treatment (5). ACC is considered a highly aggressive cancer with a high rate of recurrence and a 35% to 50% incidence of distant metastases, resulting in a low long-term survival rate (6). Surgery is currently the standard of treatment. However, surgery plus radiotherapy is the most common treatment modality, followed by surgery alone (7).

In this report, we present a case of subglottic ACC. The patient was initially diagnosed and underwent a total laryngectomy. After a 10-year period, local tumor recurrence required further surgical intervention, accompanied by postoperative radiotherapy. A total of 15 years has elapsed since the initial diagnosis and the patient is currently experiencing a recovery period, with no evidence of tumor recurrence or progression. Survival beyond 15 years for adenoid cystic carcinoma of the larynx is rarely reported in the literature.The patient’s treatment experience can serve as a valuable reference point for the diagnosis and treatment of analogous diseases. The aim of this study was to provide more information on the characterization, diagnosis, and treatment of the disease through one clinical case and a review of the literature on the subject.

## 2 Case presentation

**Figure 1:**
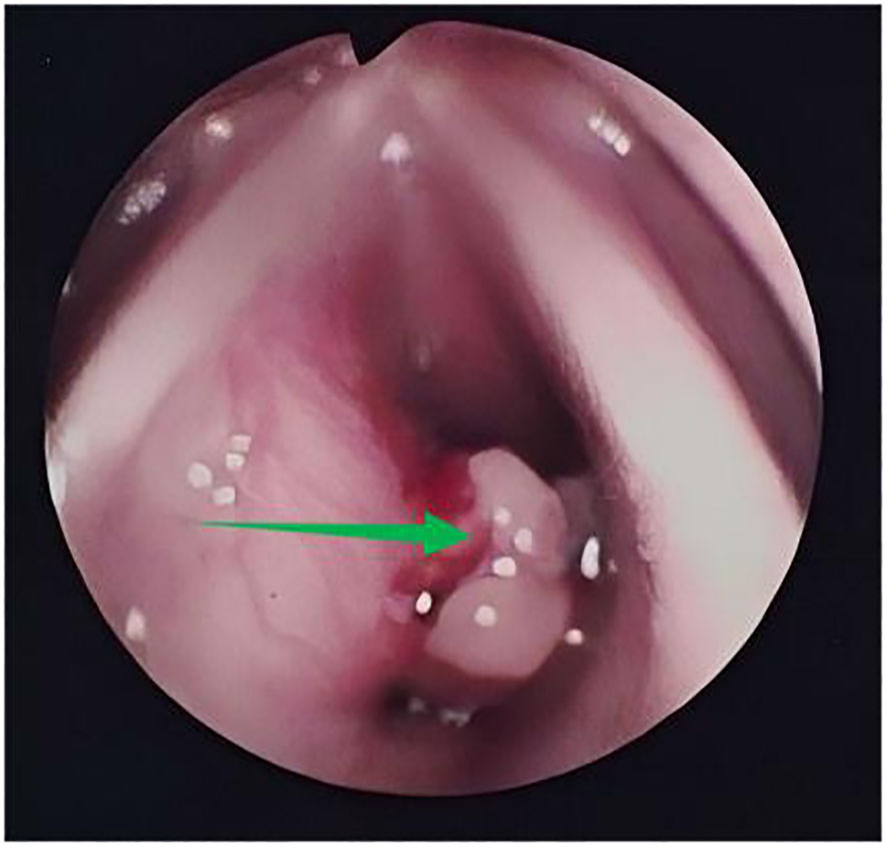
Laryngoscopy in 2010 showed a mass in the left subglottic area (green arrow).

In 2010, a 38-year-old patient with symptoms of hoarseness for 1 year, wheezing after activity for 4 months, and intermittent cough for 1 month experienced gradual worsening of symptoms. She had been treated with oral antibiotics without improvement. The patient has no medical history of smoking or heavy drinking. Nor does the patient have a family history of genetic disorders. Fiberoptic laryngoscopy showed a markedly bulging left subglottis with nodular unevenness and bleeding on palpation (**Figure 1**).

**Figure 2:**
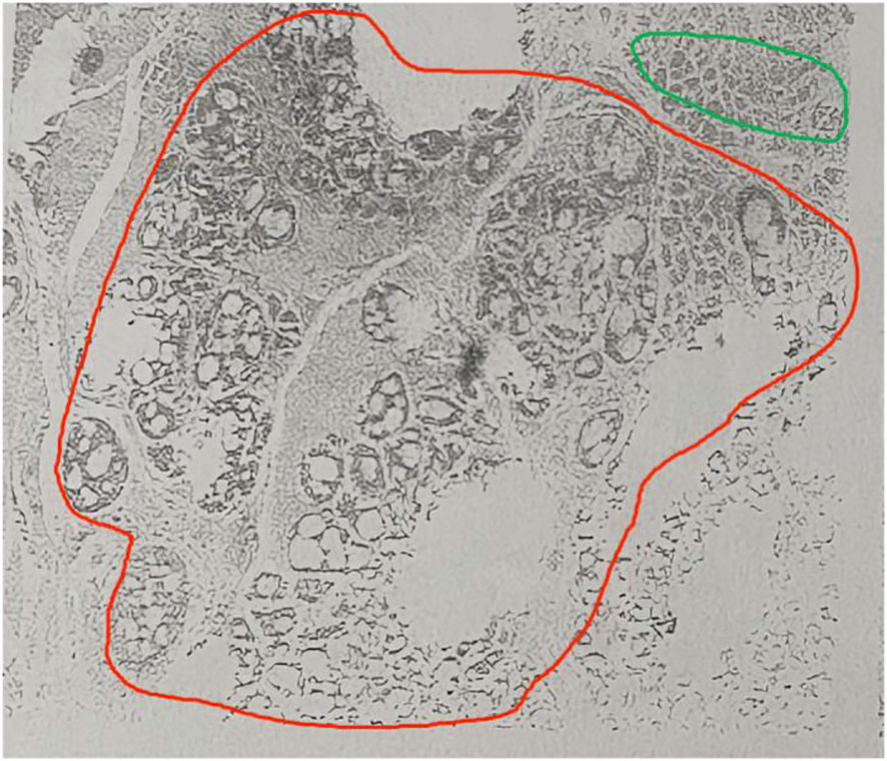
Hematoxylin/eosin staining of an adenoid cystic carcinoma. Magnification, x40. Sieve type in red circle, tube type in green circle.

The biopsy pathology revealed ACC, a rare type of salivary gland malignancy. Magnetic resonance imaging (MRI) of the neck identified a left subglottic lesion measuring approximately 2.0×1.5×1.0 cm, with no enlarged lymph nodes in the neck. A comprehensive diagnostic imaging evaluation was performed, including neck ultrasound, abdominal ultrasound and chest computed tomography (CT). However, these thorough examinations did not detect any distant neoplastic lesions. The patient underwent a total laryngectomy under general anesthesia. The subsequent postoperative pathological report showed ACC (**Figure 2**) of the left subglottic valve, the pathological margins were negative.

**Figure 3:**
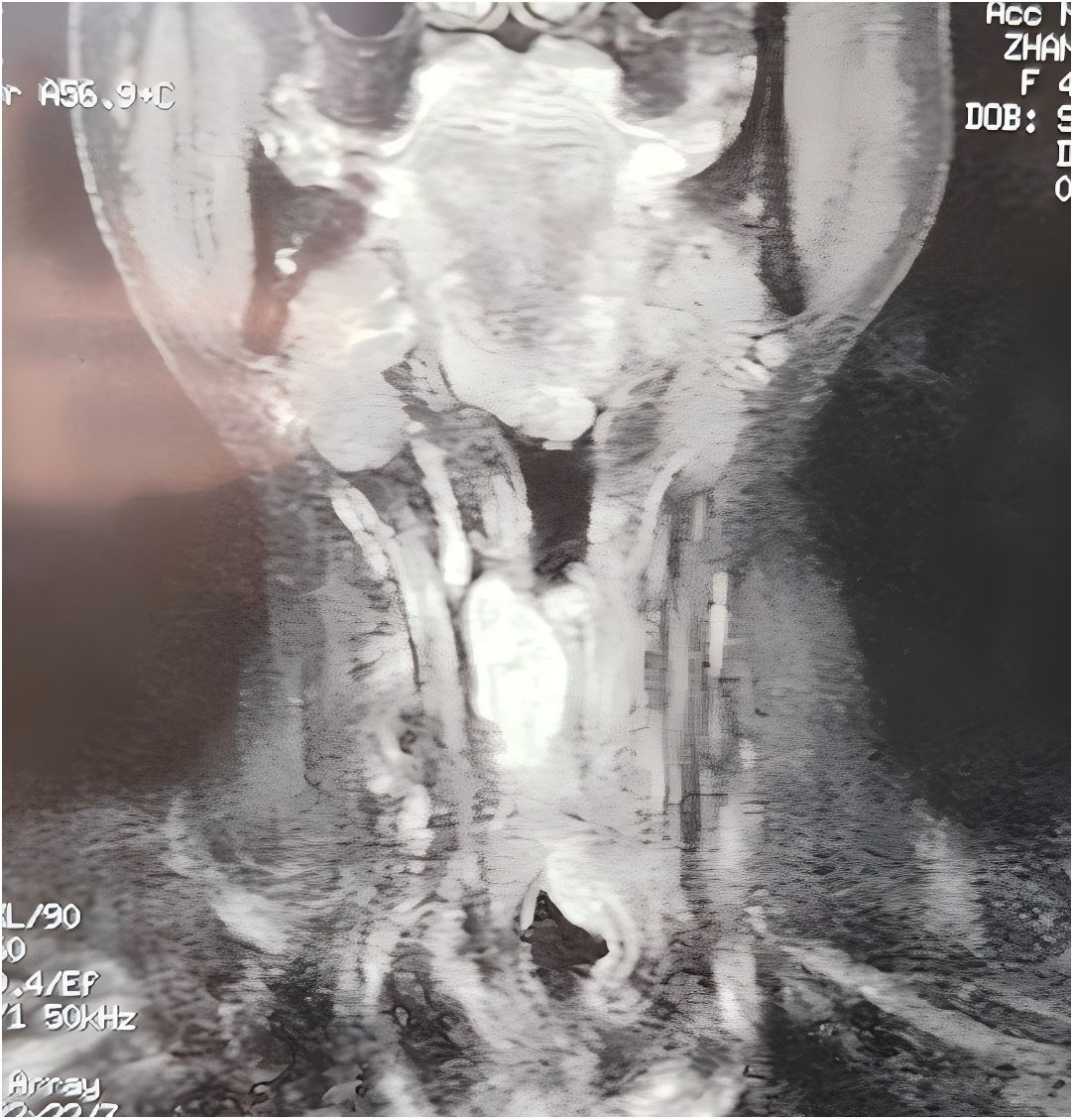
A magnetic resonance examination of the neck in 2020 showing irregular soft tissue shadows in the tracheostomy opening and in the overlying larynx, with homogeneous enhancement on enhancement, and a lesion measuring approximately 3.0×2.6×6.6 cm.

The patient recovered well after the operation, received no other treatment and was able to communicate with simple speech through training in esophageal articulation. However, a review in 2020 revealed a local recurrence of the tumor. A subsequent enhanced magnetic resonance examination of the neck revealed irregular soft tissue shadows visible in the tracheotomy and the anterior laryngeal cavity, with clearly defined borders and homogeneous confluent enhancement after enhancement. The dimensions of the lesion were approximately 3.0×2.6×6.6 cm (**Figure 3**). No enlarged lymph nodes were observed in the neck bilaterally.

The patient underwent an extended resection of a tracheostomy tumor under general anesthesia. During surgery, tumors were found to invade both the thyroid glands and the cervical esophagus. During the operation, the invaded cervical esophagus was resected, the bilateral thyroid glands were removed, and the defective cervical esophageal wall was repaired with a right supraclavicular flap. Intraoperative rapid frozen section analysis revealed negative cervical esophageal margins and negative tracheostomy margins. During the surgical procedure, multiple black lymph nodes were identified in the vicinity of the right tracheoesophageal groove. Subsequent to the removal of these lymph nodes and their examination by frozen section, no evidence of metastasis was detected. Postoperatively, the patient demonstrated a positive recovery trajectory. The gastric tube was removed 10 days after the operation. The patient required oral thyroid hormone tablets and calcium tablets.

A thorough analysis of the conventional pathology report revealed the presence of ACC, showing a mixed sieve and tubular configuration. The carcinoma showed invasive characteristics, affecting the left and right lobes of the thyroid gland and the tracheal cartilage. Furthermore, the tumor exhibited invasion of the mucosal and muscular layers of the esophagus, subcutaneous tissues, and dermis. In particular, the carcinoma also showed invasion of the neural fascia. Immunohistochemical tumor cells revealed calponin (+), CD117 (+), CK7 (+), CK8/8 (+), GFAP (-), Ki-67index (5–10%), P53 (+), P63 (+), S-100 (+), SOX-10 (+), and vimentin (+). Intraoperative frozen section pathology was negative for esophagus and fistula margins; 14 lymph nodes in the central region were without metastases.

Given the recurrence and aggressive nature of the tumor, postoperative radiotherapy was considered necessary. The patient received localized intensity modulated radiotherapy at a dose of 66 Gy in 33 sessions. During the course of radiation therapy, the patient developed grade 3 radiation mucositis and radiation dermatitis, accompanied by hypocalcemia. These conditions manifested as dry peeling of the skin on the neck, hyperpigmentation, dryness of the oropharynx, coughing, and numbness of the hands and feet. Symptomatic relief, including skin moisturizers, anti-inflammatory medications, nebulization, and calcium supplementation, were employed during the treatment regimen.

**Figure 4:**
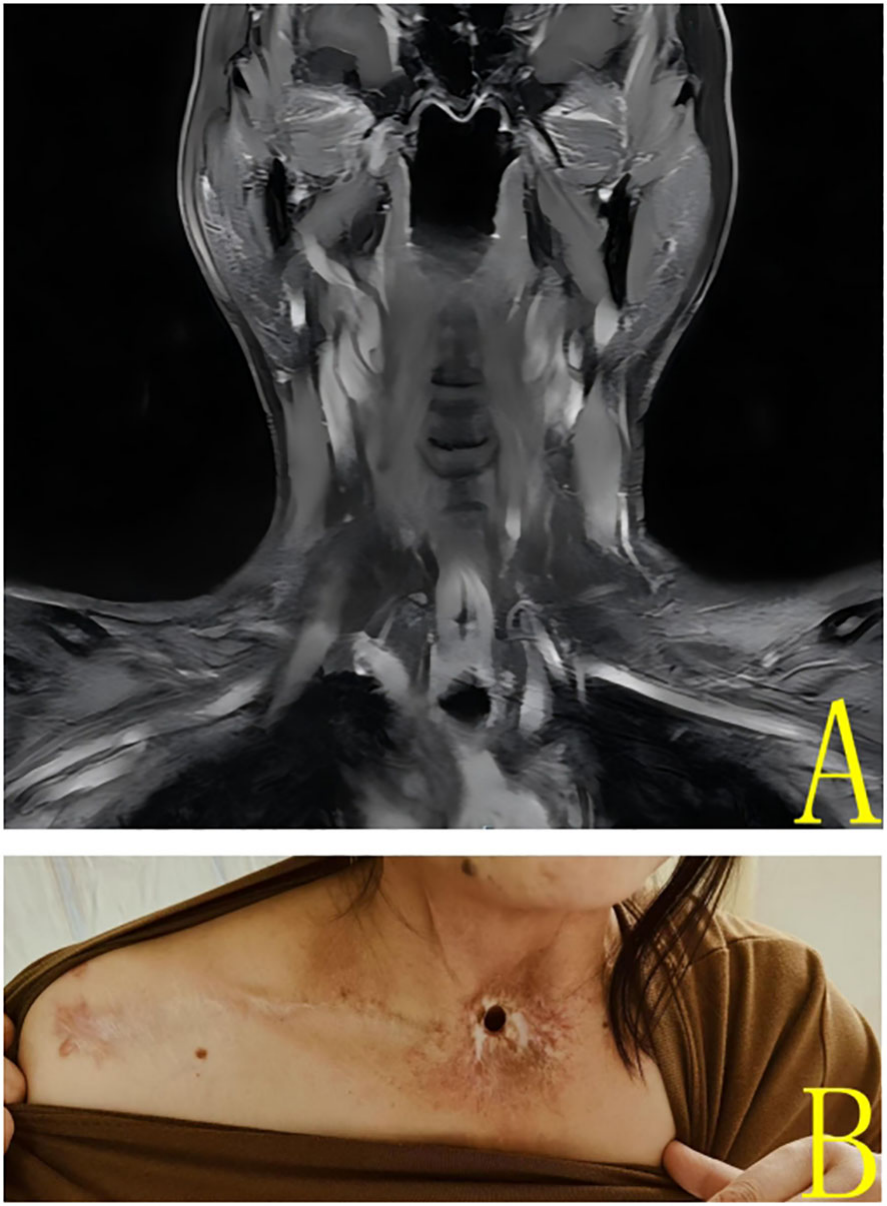
**(A)** 2025 Enhanced magnetic resonance coronal imaging of the neck showed no significant nodules or masses in the neck. **(B)** Patient’s neck photo at 2025 review.

In 2025, a further review of the patient’s case was conducted, which revealed no evidence of tumor recurrence. Neck MRI scan showed no evidence of tumor recurrence or abnormal lymph nodes (**Figure 4A**). The patient is currently undergoing a satisfactory recovery process (**Figure 4B**), despite having undergone a total laryngectomy. In particular, the patient has demonstrated the ability to communicate effectively through esophageal phonation. However, symptoms of dryness of the pharynx and intermittent coughing discomfort persisted after the end of radiotherapy.

## 3 Discussion

Approximately 90–95% of laryngeal cancers are conventional squamous cell carcinoma (SCC) and 5-10% are other histological types (8). Laryngeal ACC is very rare due to the near absence of salivary glands in the mucosa of the laryngeal-tracheal tract (0.07–0.25% of all laryngeal tumors and 1% of all ACC), most of the minor salivary glands are concentrated under the vocal folds (3). The most prevalent site of laryngeal ACC was the subglottic region (58.2%), followed by the supraglottic (32.1%) and the glottic (9.7%) regions (9). The signs and symptoms of laryngeal ACC are related to the location and size of the tumor. Subglottic tumors are more often associated with stridor and airway obstruction. Tumorous lesions in the subglottic area are rare and can be misdiagnosed as asthma, pharyngolaryngitis, and SCC of the larynx. Systematic examination of the larynx should be performed when tumors are suspected to detect lesions in the early stage and avoid omission of diagnosis or misdiagnosis. At the time of initial diagnosis, a tumor was located in the subglottic area. The patient exhibited symptoms including poor ventilation, wheezing during activity, hoarseness, and intermittent coughing.

All ages may be affected, but predominantly middle-aged individuals, with a higher incidence in women than in men (10). Unlike the more common SCC of the larynx, there is no male preference, which appears to be unrelated to smoking as a risk factor (11). This female patient was 38 years old at the time of initial diagnosis and 48 years old at the time of localized recurrence, with onset in young adulthood. The patient has no medical history of smoking or alcohol consumption. This suggests that ACC of the larynx can occur in young women, and that smoking and alcohol consumption may not be risk factors for the disease.

ACC is a biphasic tumor composed of myoepithelial and epithelial cellular components and is divided into three histological types: sieve and tubular (mostly grades 1 and 2) and solid (usually grade 3), where loss of myoepithelial cells is usually associated with invasive solid histology (12). Sieve-like is the most common histological type; the tubular type has the best prognosis; and solid tumors have the worst prognosis (13). Tumorigenic ACC cells exhibit myoepithelial and ductal differentiation and are positive for p63, S100 protein, smooth muscle actin, and cytokeratin expression (14). P53 mutation and low expression of ataxia telangiectasia mutated (ATM) protein suggest poor prognosis in ACC (15). This patient’s immunohistochemical results showed calponin (+), CD117 (+), CK7 (+), CK8/8 (+), GFAP (-), Ki-67index (5–10%), P53 (+), P63 (+), S-100 (+), SOX-10 (+), and vimentin (+). The patient’s postoperative pathology revealed ACC (mixed sieve and tubular type). The presence of tumor tissue invasion of the nerve bundle membrane was observed. The efficacy of the aggressive treatment regimen was evident in our patient. The tumor exhibited aggressive behavior, infiltrating the thyroid gland, cervical esophagus, and neural tract membrane. Following comprehensive resection and subsequent radiotherapy, the treatment outcome was satisfactory.

Surgery is the preferred treatment option and given the submucosal growth and peripheral nerve invasion of the tumor, it is widely accepted that resection should be extensive. Recurrence at the primary site was reported in 58% of patients (16). In the event of tumor recurrence, surgical intervention may be employed to excise the tumor once more. At present, surgery followed by radiotherapy is the standard treatment for ACC (17). The relative radioresistance of these tumors suggests surgical resection, which usually requires total laryngectomy due to the propensity for submucosal spread, except for perineural and lymphovascular infiltration (2).

Coca-Pelaz et al. advocated postoperative radiotherapy, which has been shown to lead to tumor regression and symptomatic relief (18). Radiotherapy is an effective treatment for ACC of the larynx with good local control rate, preserving laryngeal function and phonation, and is an important alternative to total laryngectomy (19). Fast Neutron or proton radiation therapy and carbon ion radiation therapy have been tested for ACC with satisfactory results in patients who are inoperable (20). A 16-year-old female with subglottic ACC was reported to have received proton therapy, and after 2 years of follow-up, no side effects of proton therapy were observed. In addition, the patient had no metastasis or local evidence of recurrence (21).

The use of chemotherapy in ACC remains controversial. Many studies have reported positive responses to chemotherapy and recommended it as a palliative treatment for advanced disease (22). New therapeutic approaches will focus on identifying molecular abnormalities in ACC, such as c-kit protein and epidermal growth factor receptor (EGFR) expression, in order to develop specific targeted therapies (23).

Surgery is considered essential for operable cases of head and neck ACC, for cases that are locally advanced, radiation therapy is recommended, and for cases that are at risk of distant metastasis, systemic therapy is advised (24). Upon initial diagnosis, our patient presented a tumor localized to the subglottis, and a total laryngectomy was performed. Given that the tumor had been completely excised, no adjuvant therapy was administered postoperatively. However, the tumor recurred after a decade, with invasion into surrounding structures. A second complete resection was conducted, followed by adjuvant radiotherapy, which yielded satisfactory results.Surgery plus radiotherapy and surgery alone were the most commonly used treatments, both of which resulted in approximately half of the patients surviving with no evidence of disease recurrence at follow-up, with a mean follow-up time of 54.0 months (25).

Radiation dermatitis is a common complication of radiation therapy for malignant tumors, which is mainly manifested as skin atrophy, pigmentation, delayed ulceration, and skin fibrosis. It causes significant psychological and physical distress to patients, adversely impacting their quality of life. Treatment with glucocorticoid hormone has good efficacy in preventing radiation dermatitis. Cream is a commonly used drug to prevent or reduce the effects of radiation dermatitis.Following radiation therapy the patient developed radiolucent mucositis and radiodermatitis, and these symptoms were relieved with treatment that included skin moisturizers, hormonal anti-inflammatory medications, and nebulized treatments.

Differences in survival across different patient series in previous reports may be related to differences in treatment or may be due to differences in the number of patients in each series. Ciccolallo et al. described 199 cases of ACC of the larynx and trachea, with a 5-year relative survival rate of 56.8% and a 10-year relative survival rate of 35.6% (26). Moukarbel et al. reported disease-specific 5- and 10-year survival rates of 69% and 49%, respectively, in a group of 15 cases (11). A meta-analysis of the literature on ACC of the larynx found local recurrence rates of 13.3%, regional metastasis rates of 7.8%, and distant metastasis rates of 33.3% (25).

Another feature of ACC is the low frequency of lymph node metastasis, with an overall reported incidence of approximately 10%, although this frequency may vary depending on the site of the lesion, for instance, the root of the tongue (19.2%), mobile tongue (17.6%), and floor of the mouth (15.3%) (27). Laryngeal ACC has a low rate of regional metastasis, and cervical lymphadenectomy should be performed when clinically or radiologically positive (11). In this case, no obvious cervical lymph node metastasis was detected on physical examination and radiology presented no evidence of distant metastasis. During surgery, blackened lymph nodes were identified in the right cervical region and were fully resected. Postoperative routine pathological examination of the 14 excised lymph nodes showed no evidence of metastasis. This indicates that cervical lymph node metastasis is relatively rare in laryngeal ACC.

This study has a number of limitations. First, the results of this single case and in a young patient cannot be considered generalizable. Second, the patient underwent total laryngectomy after initial diagnosis, which had a good outcome but resulted in loss of laryngeal function. In the future, the collection of additional similar cases and the evaluation of the therapeutic effects after different treatment options are needed to define the best treatment plan. Our future research will focus on effective control the tumor while preserving the laryngeal function.

Few studies in the literature are available and the follow-up period reported is relatively short. Cui et al. reported three cases of subglottic ACC with a maximum follow-up of 6 years, with one patient dying of pulmonary metastases after 6 years (28). Li et al. reported two cases of ACC of the larynx with a maximum survival of 34 months after surgery (6). Long-term follow-up is needed to better define treatment strategies and to improve tumor outcomes. Our patient was satisfied with the results of the treatment and is still in good condition after 15 years.

## 4 Conclusion

Subglottic ACC is very rare and usually presents as locally advanced and aggressive disease. Here, we describe a case of subglottic ACC, accompanied by a detailed account of the patient’s surgical intervention, subsequent reoperation due to recurrence, and postoperative radiotherapy. Early diagnosis and complete surgical resection can lead to favorable outcomes; the patient has survived long-term. In the event of local recurrence, effective treatment strategies include surgical intervention and postoperative radiotherapy. The accelerated development of novel pharmacotherapeutic agents in the forthcoming years may facilitate enhanced control and efficacy of targeted therapy.

## Data Availability

The raw data supporting the conclusions of this article will be made available by the authors, without undue reservation.
